# Environmental enrichment is associated with favorable memory-related functional brain activity patterns in older adults

**DOI:** 10.3389/fnagi.2024.1451850

**Published:** 2024-12-24

**Authors:** Simon Hass, Maxie Liebscher, Anni Richter, Klaus Fliessbach, Christoph Laske, Sebastian Sodenkamp, Oliver Peters, Julian Hellmann-Regen, Ersin Ersözlü, Josef Priller, Eike Jakob Spruth, Slawek Altenstein, Sandra Röske, Anja Schneider, Hartmut Schütze, Annika Spottke, Anna Esser, Stefan Teipel, Ingo Kilimann, Jens Wiltfang, Ayda Rostamzadeh, Wenzel Glanz, Enise I. Incesoy, Falk Lüsebrink, Peter Dechent, Stefan Hetzer, Klaus Scheffler, Michael Wagner, Frank Jessen, Emrah Düzel, Franka Glöckner, Björn Hendrik Schott, Miranka Wirth, Olga Klimecki, for DELCODE study group

**Affiliations:** ^1^German Center for Neurodegenerative Diseases (DZNE), Dresden, Germany; ^2^Leibniz Institute for Neurobiology, Magdeburg, Germany; ^3^German Center for Mental Health (DZPG), partner site Halle-Jena-Magdeburg, Magdeburg, Germany; ^4^Center for Intervention and Research on Adaptive and Maladaptive Brain Circuits Underlying Mental Health (CIRC), Halle-Jena-Magdeburg, Magdeburg, Germany; ^5^German Center for Neurodegenerative Diseases (DZNE), Bonn, Germany; ^6^Department of Old Age Psychiatry and Cognitive Disorders, University Hospital Bonn and University of Bonn, Bonn, Germany; ^7^German Center for Neurodegenerative Diseases (DZNE), Tübingen, Germany; ^8^Section for Dementia Research, Department of Psychiatry and Psychotherapy, Hertie Institute for Clinical Brain Research, University of Tübingen, Tübingen, Germany; ^9^Department of Psychiatry and Psychotherapy, University of Tübingen, Tübingen, Germany; ^10^German Center for Neurodegenerative Diseases (DZNE), Berlin, Germany; ^11^Department of Psychiatry and Neuroscience, Charité - Universitaetsmedizin Berlin, Berlin, Germany; ^12^ECRC Experimental and Clinical Research Center, Charité - Universitaetsmedizin Berlin, Berlin, Germany; ^13^Department of Psychiatry and Psychotherapy, Charité - Universitaetsmedizin Berlin, Berlin, Germany; ^14^Department of Psychiatry and Psychotherapy, School of Medicine, Technical University of Munich, Munich, Germany; ^15^UK DRI, University of Edinburgh, Edinburgh, United Kingdom; ^16^German Center for Neurodegenerative Diseases (DZNE), Magdeburg, Germany; ^17^Department of Neurology, University of Bonn, Bonn, Germany; ^18^German Center for Neurodegenerative Diseases (DZNE), Rostock, Germany; ^19^Department of Psychosomatic Medicine, Rostock University Medical Center, Rostock, Germany; ^20^German Center for Neurodegenerative Diseases (DZNE), Göttingen, Germany; ^21^Department of Psychiatry and Psychotherapy, University Medical Center Göttingen, University of Göttingen, Göttingen, Germany; ^22^Neurosciences and Signaling Group, Department of Medical Sciences, Institute of Biomedicine (iBiMED), University of Aveiro, Aveiro, Portugal; ^23^Department of Psychiatry, Medical Faculty, University of Cologne, Cologne, Germany; ^24^Institute of Cognitive Neurology and Dementia Research (IKND), Otto-von-Guericke University, Magdeburg, Germany; ^25^Department for Psychiatry and Psychotherapy, University Clinic Magdeburg, Magdeburg, Germany; ^26^MR-Research in Neurosciences, Department of Cognitive Neurology, Georg-August-University Göttingen, Göttingen, Germany; ^27^Berlin Center for Advanced Neuroimaging, Charité – Universitaetsmedizin Berlin, Berlin, Germany; ^28^Department for Biomedical Magnetic Resonance, University of Tübingen, Tübingen, Germany; ^29^Excellence Cluster on Cellular Stress Responses in Aging-Associated Diseases (CECAD), University of Cologne, Cologne, Germany; ^30^Chair of Behavioral Psychotherapy, Institute for Clinical Psychology and Psychotherapy, Dresden University of Technology, Dresden, Germany; ^31^Faculty of Biopsychology, Dresden University of Technology, Dresden, Germany

**Keywords:** memory network, subjective cognitive decline, multimodal leisure activities, prevention, dementia

## Abstract

**Background:**

In humans, environmental enrichment (EE), as measured by the engagement in a variety of leisure activities, has been associated with larger hippocampal structure and better memory function. The present cross-sectional study assessed whether EE during early life (13–30 years) and midlife (30–65 years) is associated with better preserved memory-related brain activity patterns in older age.

**Methods:**

In total, 372 cognitively unimpaired older adults (aged ≥60 years old) of the DZNE-Longitudinal Study on Cognitive Impairment and Dementia (DELCODE; DRKS00007966) were investigated. EE was operationalized using items of the Lifetime of Experiences Questionnaire (LEQ), which measures the self-reported participation in a variety of leisure activities in early life and midlife. The preservation of memory-related functional brain activity was assessed using single-value scores, which relate older adults’ brain activity patterns in the temporo-parieto-occipital memory network to those of young adults during visual memory encoding (FADE and SAME scores).

**Results:**

EE during early life and midlife was significantly associated with higher SAME scores during novelty processing (*n* = 372, *β* = 0.13, *p* = 0.011). Thus, older participants with higher EE showed greater similarity of functional brain activity patterns during novelty processing with young adults. This positive association was observed most strongly in participants with subjective cognitive decline (SCD, *n* = 199, *β* = 0.20, *p* = 0.006).

**Conclusion:**

More frequent participation in a variety of leisure activities in early life and midlife is associated with more successful aging of functional brain activity patterns in the memory network of older adults, including participants at increased risk for dementia. Longitudinal studies are needed to clarify whether higher EE during life could help preserve memory network function in later life.

## 1 Introduction

A significant proportion of the risk for aging-related conditions, including dementia, can be attributed to modifiable factors (Gatz et al., 2006; Livingston et al., 2024). It has been estimated that up to 40% of dementia cases could be prevented or delayed by modifying environmental influences (Nichols et al., 2022). Thus, simultaneously addressing the multiple modifiable risk factors for dementia, including loneliness, as well as physical and cognitive inactivity, can promote healthy aging and reduce the risk of dementia considerably (Livingston et al., 2024).

In humans, environmental factors may include leisure activities (Kempermann, 2022), analogous to the concept of environmental enrichment, which has been extensively studied in animal models (Kempermann, 2019). In animal models, environmental enrichment has been associated with greater plasticity of memory-related brain structure and function (Kempermann et al., 2010; Kempermann, 2019). The relationship between environmental enrichment, as measured by the engagement in a variety of leisure activities across the lifespan, and the preservation of memory network function in later life has not been investigated to the same extent in humans. In the present study, we aimed to address this gap by investigating whether greater environmental enrichment during early life and midlife would be associated with better preserved memory-related functional brain activity in older participants from the multi-center DZNE-Longitudinal Study on Cognitive Impairment and Dementia (DELCODE; Jessen et al., 2018).

### 1.1 Environmental enrichment

Environmental enrichment in terms of motor, cognitive, sensory, and social stimulation has been shown to elicit long-term positive effects on the brain in animal models (Fabel et al., 2009; Kempermann, 2019; Manno et al., 2022). More specifically, an enriched environment in rodents (as opposed to standard housing conditions) has been shown to induce structural and functional changes in the hippocampus (Manno et al., 2022), promote cognitive flexibility (Zeleznikow-Johnston et al., 2017) as well as spatial memory (Frick et al., 2003), and even improve declarative memory in offspring exposed to alcohol during development (Brancato et al., 2020). The present study employed a translational perspective: we based our theoretical model and research objectives on the findings from animal studies that have made significant progress in the field of environmental enrichment, and applied the concept to humans.

In humans, environmental enrichment can be characterized by motor, cognitive, sensory, and social stimulations as facilitated by participation in a variety of leisure activities across the lifespan. Leisure activities are activities that people tend to pursue in their free time and that affect a person’s perceived quality of life in different ways (Brajša-Žganec et al., 2011). Engagement in a variety of leisure activities (or a life rich in stimuli and activities), subsequently referred to as environmental enrichment in humans, can be assessed using the Lifetime of Experiences Questionnaire (LEQ; Valenzuela and Sachdev, 2007), which quantifies environmental enrichment by assessing the frequency of participation in social, musical, artistic, cognitive and physical leisure activities across the lifespan. The LEQ represents a highly useful tool for the measurement of cognitive reserve and resilience (Kartschmit et al., 2019). The LEQ (and its subscales) have been evaluated in multiple studies of older cohorts (Chan et al., 2018; Ourry et al., 2021; Karsazi et al., 2022), including the DELCODE cohort (Ballarini et al., 2023; Ersoezlue et al., 2023; Klimecki et al., 2023).

### 1.2 Environmental enrichment and the human brain

The existing literature in humans suggests an association between environmental enrichment in terms of engagement in leisure activities and more favorable outcomes in brain and neurocognitive aging (Hertzog et al., 2008). This is particularly true for the association between environmental enrichment factors and structural brain integrity in humans. For example, cross-sectional studies found that higher LEQ scores were associated with increased gray matter volume, especially in the hippocampus (Suo et al., 2012), and preserved white matter microstructure of the memory system in older adults (Klimecki et al., 2023). In addition, higher self-reported cognitive and physical activities have been associated with fewer white matter lesions in our previous study in older adults (Wirth et al., 2014), as well as preserved brain volume and greater plasticity of neural circuits (for a review, see Cheng, 2016). A longitudinal study conducted in older adults showed that three-year changes in white matter microstructure accounted for the association between changes in self-reported leisure activities (predominantly social activities) and changes in perceptual speed (Köhncke et al., 2016).

In contrast, research on the impact of leisure activities on functional brain activity is scarce. Predominantly, effects of physical activity on functional brain connectivity (Anatürk et al., 2021) or functional brain activity (Domingos et al., 2021) have been investigated. Older adults with higher mean physical activity during an 18-month period showed greater blood oxygen level-dependent (BOLD) responses in several brain structures, including the medial frontal gyrus and precuneus, during a semantic memory task (Smith et al., 2016). Furthermore, older adults who engaged in aerobic physical activities showed greater BOLD responses in the left hippocampus during memory encoding (Thielen et al., 2016). In contrast, studies on the association between a variety of leisure activities on functional brain activity in the memory network of older adults are still missing.

### 1.3 The present study

To address this gap, the present cross-sectional study used a sample of cognitively unimpaired older adults from the DELCODE study (Jessen et al., 2018) to investigate whether greater environmental enrichment during early life and midlife is associated with a better preservation of memory-related functional brain activity in late life. The following methodological approach has been taken to examine our research question:

We operationalized human environmental enrichment using items of the LEQ (Valenzuela and Sachdev, 2007), which quantify the self-reported frequency of engagement in a variety of leisure activities across the lifespan. A similar procedure with an overlapping sample was used in our previous study on fornix microstructure (Klimecki et al., 2023). Memory-related functional brain activity was operationalized using single-value scores, which quantify Functional Activity Deviation during Encoding (FADE) and the Similarity of Activations during Memory Encoding (SAME) in relation to a reference sample of healthy young adults. The FADE and SAME scores were derived for any given participant of the DELCODE cohorts using a functional magnetic resonance imaging (fMRI) paradigm on visual memory encoding (Soch et al., 2021). Both FADE and SAME scores can be interpreted as biomarkers for successful aging of the memory network, as they are computed from novelty-related and memory-related activation patterns in the temporo-parieto-occipital memory network (Düzel et al., 2010; Soch et al., 2021, 2024; Richter et al., 2023). Previous studies have demonstrated that these scores predict explicit memory performance in healthy older adults (Soch et al., 2022) and are associated with clinical and biochemical indices of risk for Alzheimer’s disease (Soch et al., 2024).

We hypothesized that more frequent participation in a variety of leisure activities during life would be associated with better preservation of memory-related functional brain activity patterns in older adults. This was assessed using the FADE and SAME scores, which measure the similarity of memory-related functional brain activity patterns in any given older participant to those observed in young adults. Specifically, we expected that greater environmental enrichment would be associated with lower FADE scores (i.e., indicating less deviation from functional activity patterns observed in young adults) as well as higher SAME scores (i.e., indicating greater similarity with functional activation and deactivation patterns observed in young adults).

## 2 Methods

### 2.1 Participants

All participants were recruited from the baseline dataset of the multi-center DELCODE study that was in detail described previously (Jessen et al., 2018). The DELCODE study was designed according to the ethical principles of the Declaration of Helsinki. Local ethical committees at each participating study site approved the study protocol. All participants gave written informed consent. The DELCODE study was registered at the German Clinical Trials Register (DRKS00007966; date: 2015/05/04).

Exclusion and inclusion criteria of the DELCODE study are provided elsewhere (Jessen et al., 2018). In brief, participants had to meet the criteria of being 60 years of age or older and of speaking German fluently. For the DELCODE study, participants were either (self-) referred to or recruited via local newspaper advertisements into various diagnostic subgroups, including cognitively unimpaired older adults (OA), participants with subjective cognitive decline (SCD), participants with diagnosed Alzheimer’s disease (AD) or mild cognitive impairment (MCI), and participants with a family history of AD (FH). Baseline assessments comprised a clinical and risk factor assessment (which also included the LEQ), a neuropsychological test battery, biomaterial sampling, cerebrospinal fluid AD biomarker assessment, as well as structural and functional MRI (Jessen et al., 2018).

**Figure 1:**
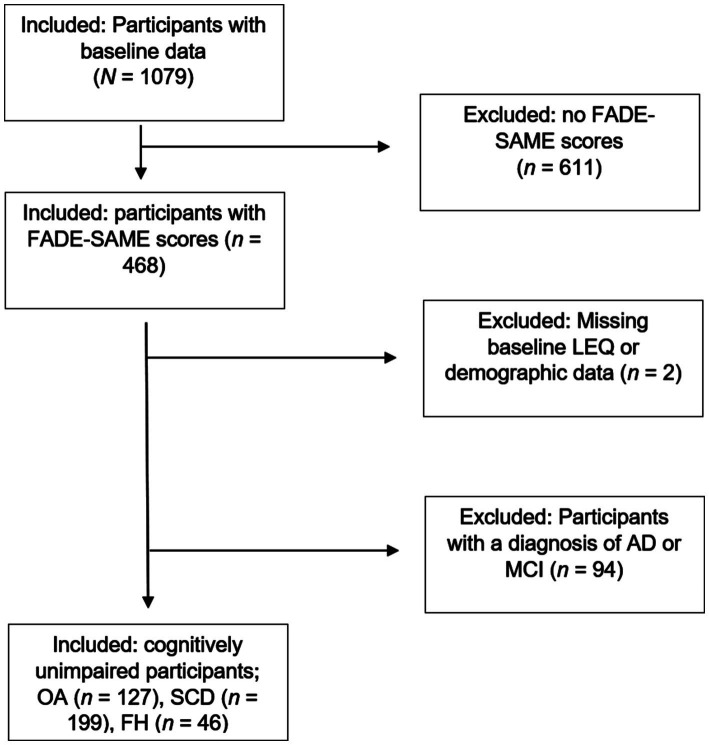
Process of participant selection. The initial sample size of the DELCODE baseline dataset (data release for this study: 01.2021) was *N* = 1,079. Out of these, *n* = 468 had complete MRI data and thus FADE-SAME scores available. After exclusion of two participants with missing baseline LEQ and demographic data and 94 participants with a diagnosis of AD or MCI, the final sample amounted to *n* = 372 participants with 197 women and 175 men and the following subgroups: *n* = 127 OA, *n* = 199 participants with SCD, and *n* = 46 participants with FH. FADE/SAME, functional activity deviation during encoding/similarity of activations during memory encoding; LEQ, Lifetime of Experiences Questionnaire; AD, Alzheimer’s Dementia; MCI, mild cognitive impairment; OA, cognitively unimpaired older adults; SCD, subjective cognitive decline; FH, family history of AD.

The present study focused on identifying potential associations between environmental enrichment and successful aging of functional brain activity patterns in the human memory network *before* the manifestation of objective cognitive impairment. We therefore chose to exclude participants diagnosed with objective cognitive impairment (MCI or AD) to tighten the focus on healthy brain aging. By including only participants within the cognitively normal range from the following subgroups, OA, participants with SCD, and participants with FH, we aimed to better understand the processes underlying healthy brain aging, free from confounding effects of clinical symptoms. In total, data of 372 healthy, cognitively unimpaired participants of the DELCODE study were included in our statistical analyses. A sample size of 372 should allow us to detect moderate-to-large effects accurately. The extensive selection flow chart is depicted in Figure 1.

### 2.2 Materials

#### 2.2.1 Lifetime of experiences questionnaire

The LEQ (Valenzuela and Sachdev, 2007) was used to measure environmental enrichment. The LEQ is an established questionnaire that has been translated and validated in many languages (Ourry et al., 2021) including German (Roeske et al., 2018) and has been implemented in related studies (e.g., Klimecki et al., 2023).

Among others, the self-reported questionnaire quantifies the frequency of participation in physical, cognitive, sensory, and social leisure activities across the lifespan. The LEQ itself has been constructed alongside two dimensions: Life stages that include three subsequent life periods of early life (13–30 years), midlife (30–65 years), and late life (65 + years), and specific/non-specific mental activities that assess lifestyle activities an individual had engaged in within each of the three life stages. The specific life stage questions mainly relate to the extent and nature of educational and occupational experiences across life. The non-specific life stage questions are a validated measure of leisure activity engagement across life (Valenzuela and Sachdev, 2007) and relate to an individual’s frequency of participation in six leisure activities, namely social activity, musical activity, artistic activity, physical activity, reading (as a proxy for cognitive activity), and speaking any additional language. The non-specific items are assessed for each life stage in the LEQ, i.e., for early life (13–30 years), midlife (30–65 years), and late life (65 + years).

For this study’s purpose, we used the non-specific LEQ questions or subscales (in terms of frequency of engagement in leisure activities) across two life stages of the German version of the LEQ (Roeske et al., 2018). Namely, participants were asked to indicate how frequently they (had) engaged in the aforementioned six leisure activities retrospectively for two specific life stages (13–30 and 30–65 years, respectively). The scoring was done using a 6-point Likert scale: A daily participation in any activity corresponds to five points, weekly participation to four points, participation twice per month to three points, once per month to two points, less than once per month to one point, and never having participated to zero points.

Next, we summed up the points assigned for the frequency of participation across the two life stages (Note, only items concerning the two life stages 13–30 and 30–65 years have been considered since 103 participants were younger than 65). As a result, a maximum score of 30 could be achieved for each of the two specific life stages (i.e., the maximum being five points for any of the six leisure activities). In the LEQ, non-specific life stage questions also comprised an item related to traveling outside of Germany. This item was disregarded in the present study, since the traveling restrictions in the former German Democratic Republic may have biased the self-reports.

#### 2.2.2 Functional MRI data analysis

The FADE and SAME scores (Düzel et al., 2010; Soch et al., 2021) were obtained from fMRI data measured during a visual memory encoding experiment. The fMRI task has been described in detail in previous publications (Düzel et al., 2010; Soch et al., 2021). Specifics regarding the acquisition and processing of fMRI data can be found in Supplementary material A.

In brief, within the incidental encoding task, participants had to categorize whether photographs depicted indoor or outdoor scenes. Participants viewed 88 novel photographs (44 indoor and 44 outdoor scenes) as well as repetitions of two highly familiar “master” photographs they had been familiarized with before the actual experiment, using 10 repetitions directly before MR scanning (Düzel et al., 2010). The two master photographs consisted of one indoor and one outdoor photograph, which were shown 22 times each during fMRI, resulting in 22 indoor and 22 outdoor trials. After having completed the fMRI session, participants performed a recognition memory test on a computer outside of the scanner. Here, participants were presented with the same 88 photographs they had seen in the fMRI visual memory encoding phase (thus, the previously novel photographs would be regarded as “old” stimuli now) and 44 photographs they had not seen before (thus, “new” stimuli). Participants then rated each photograph according to their confidence of recalling it on a five-point Likert scale ranging from 1 (“definitely new”) to 5 (“definitely old”) (Düzel et al., 2010; Soch et al., 2021).

For the calculation of the FADE and SAME scores, two different contrasts were taken into account: Novelty processing (novel vs. master stimuli) and subsequent memory (novel images as a function of later recognition). The subsequent memory contrast was based on the participant’s recognition-confidence ratings which they indicated on a five-point Likert scale during retrieval. Essentially, BOLD responses to novel photographs in the encoding phase were parametrically modulated with the recognition-confidence ratings the participants submitted for the “old” photographs after the fMRI session (Soch et al., 2021). Interrogating the subsequent memory contrast by testing the parametric modulator regressor therefore reflects the brain activity associated with subsequent remembering of previously presented items, i.e., activation differences between successful memory encoding (reflected by high confidence ratings 4–5) and forgetting (reflected by low confidence ratings 1–2). Novelty processing was assessed via a differential contrast comparing the neural response to novel vs. familiar photographs in the fMRI session (Richter et al., 2023).

### 2.3 Outcome measures

#### 2.3.1 Environmental enrichment

Environmental enrichment was operationalized using numerical single-value scores derived from the non-specific questionnaire items of the LEQ (Roeske et al., 2018). The two subscores reflecting the frequency of participation in the six leisure activities (namely, social activity, musical activity, artistic activity, physical activity, reading, and speaking an additional language) within the two distinct life stages (i.e., 13–30 and 30–65 years) were added up to constitute a numerical single-value score for each participant, the environmental enrichment score.

#### 2.3.2 Memory-related functional brain activity

Memory-related functional brain activity was operationalized using the FADE and SAME scores extracted from fMRI data (Düzel et al., 2010; Soch et al., 2021). Both FADE and SAME scores can be interpreted as measures of successful aging of the memory network by comparing an older adult’s brain activation pattern during novelty processing and memory encoding to average patterns seen in young adults in the temporo-parieto-occipital memory network. FADE and SAME scores have been robustly associated with measures of episodic and working memory, alertness, speed, and cognitive flexibility in older adults (Richter et al., 2023).

**Figure 2:**
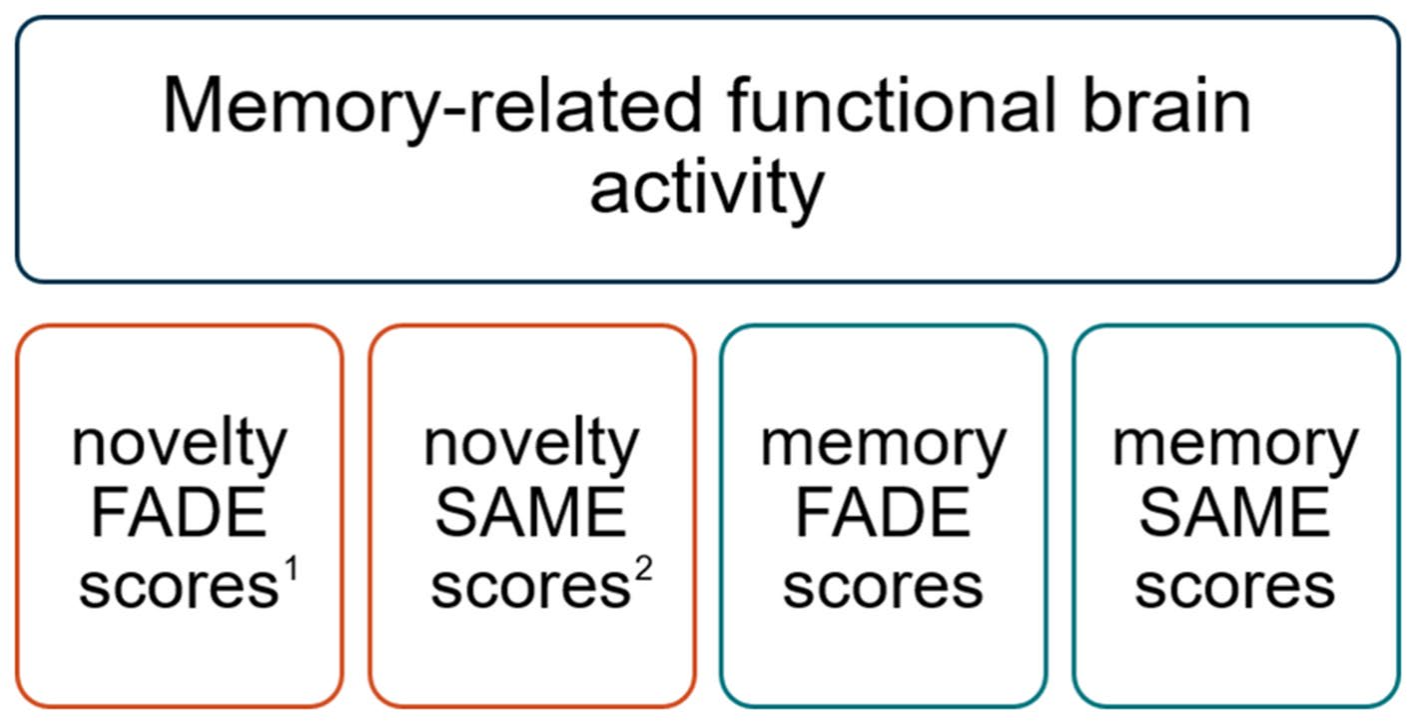
Comprehensive overview of the FADE-SAME scores. ^1^FADE scores reflect the deviation of functional brain activity during novelty processing and memory encoding. ^2^SAME scores reflect the similarity of activations (and deactivations) during novelty processing and memory encoding. FADE and SAME scores are inversely correlated, since the FADE score captures the deviation of functional activity patterns, whereas the SAME score captures the similarity of functional activity patterns. However, the SAME score also quantifies reduced deactivations, most prominently in default mode network (DMN) regions. Thus, FADE and SAME scores are rather complementary measures of successful aging in memory seen with respect to young adults (Soch et al., 2021; Richter et al., 2023). FADE, functional activity deviation during encoding; SAME, similarity of activations during memory encoding.

The extraction of FADE-SAME scores for each participant of the DELCODE study has been described recently (Soch et al., 2024) and is summarized in Supplementary material A3. For each contrast, novelty processing and subsequent memory, contrast maps from older subjects were analyzed in relation to group contrasts from a sample of 106 young, healthy adults (for details about this cohort, see Soch et al., 2021), showing positive effects (“activations”) or negative effects (“deactivations”) in the respective contrast within the temporo-parieto-occipital memory network. The FADE score reflects the mean t-value of an older participant’s contrast in all voxels in which young participants show a positive effect on this contrast, subtracted from the mean t-value of the same contrast outside those voxels (Soch et al., 2021, 2024). The SAME score is computed as the sum of (i) the mean of an older individual’s reduced activations in all voxels in which young adults show a positive effect and (ii) the mean of reduced deactivations in all voxels with a negative effect in the young reference sample. In addition to their opposite direction (similarity vs. difference), SAME scores differ from FADE scores (i) by considering both, functional activation *and* deactivation patterns in the respective contrast, and (ii) by accounting for the variability of fMRI activity within the reference sample of young subjects (Soch et al., 2021). See Figure 2 for a comprehensive overview of the FADE-SAME scores.

### 2.4 Data analyses

#### 2.4.1 Data preparation and processing

Data processing was carried out using R 4.2.1 (R Core Team, 2022) in RStudio (version 2023.12.1.402; Posit Team, 2024). *N* = 30 participants were lacking LEQ data on a varying number of items (ranging from 1 to 12 missing items), with the missing values being distributed roughly equally across the two life stages. We performed the Little’s MCAR test (Little, 1988) to ascertain the missing values were *missing completely at random* (MCAR), i.e., not showing any particular pattern of missingness which would entail biases. If the missing data is unrelated to any variables in the data set, it can safely be considered a random sample of the complete data, and the implicated loss of power can be recovered (Little et al., 2014).

According to the MCAR test (Little, 1988), the missing data in the present study was *missing completely at random* (*Χ*^2^ = 13.46, *p* = 0.891). Hence, we proceeded with the Full Information Maximum Likelihood (FIML; Peters and Enders, 2002) method to obtain regression parameter estimates in the presence of missing data. Assuming the missing data is MCAR, maximum likelihood methods produce unbiased estimates and accurate standard errors, thus constituting recommended techniques (Newman, 2014). The FIML method directly estimates the parameters of interest from an incomplete data matrix (Graham, 2009). In our case, regression coefficients describing effects of environmental enrichment and demographic variables on FADE and SAME scores were estimated. The FIML regressions were performed using the *lavaan* package in R (version 0.6.17; Rosseel, 2012).

#### 2.4.2 Measurement of covariates

We assumed that the association between environmental enrichment during early life and midlife and memory-related functional brain activity might be affected by the age, sex, educational level and socioeconomic status of the participants. These measures were included in the data release from the DELCODE study. The respective age (i.e., years of age) of the participants was calculated with the R package *eeptools* (version 1.2.5; Knowles, 2020) since the data release primarily included birth year and birth month. Sex was coded as a binary variable (female/male) in the DELCODE cohort. Educational level was assessed in years of education. Socioeconomic status (SES) was estimated using the International Socioeconomic Index of Occupational Information (ISEI; Ganzeboom et al., 1992) ranging from 16 to 90, with higher scores corresponding to a higher socioeconomic status. The ISEI calculation was done using the self-reported occupational history assessed by the LEQ and an automated crosswalk-cased calculation procedure, as described in detail in our previous study (Böttcher et al., 2022).

#### 2.4.3 Statistical analyses

All data analyses and the respective results can be retraced in the R Markdown files available in the Open Science Framework: https://osf.io/amgv2/. Confirmatory analyses included regression analyses performed in R 4.2.1 (R Core Team, 2022) in RStudio (version 2023.12.1.402; Posit Team, 2024) with *lavaan* (Rosseel, 2012). We assessed the hypothesis that the environmental enrichment score would be associated with memory-related functional brain activity patterns, as operationalized via FADE-SAME scores. Prior to conducting the regression analysis, regression assumptions were assessed using the *ILSE* package (version 1.1.7; Liu et al., 2022) to extract residuals: Normality was assumed based on the central limit theorem and checked with Q-Q plots, linearity was examined with residuals vs. predicted values plots, and homoscedasticity with scale-location plots.

Regression analysis in the entire sample: We first performed separate simple linear regression analyses with the FIML method in *lavaan* (Rosseel, 2012) with 4 FADE-SAME scores (2 scores from the novelty contrast and 2 scores from the memory contrast) as dependent variables and the environmental enrichment score as independent variable. All variables used in the FIML regression analyses were scaled in advance in order to obtain standardized beta coefficients. The directionality and statistical significance of the respective associations was determined with beta- and *p*-values provided by the FIML method. We further assessed whether auxiliary variables would enhance the predictive accuracy of the regression models using the package *semTools* (version 0.5.6; Jorgensen et al., 2022). Note that, since the inclusion of auxiliary variables did not yield substantially better FIML regression models, we only report the FIML regression models without auxiliary variables.

Exploratory analysis within subgroups: since past research has suggested that SCD is associated with increased risk for developing MCI and dementia (Jessen et al., 2020), we sought to confirm if all three participants subgroups (OA, SCD, and FH) would feature expected associations between environmental enrichment scores and memory-related functional brain activity. To explore this, we performed simple linear regression analyses separately for each of the three subgroups. This within-group regression analysis was conducted with those FADE and SAME scores that showed a significant association in the entire sample as dependent variable and environmental enrichment as independent variable. All variables used in the FIML regression analysis were separately scaled within each subgroup to reflect subgroup-specific variations. In a *post-hoc* exploratory analysis, we further compared the regression slopes for the three subgroups by inserting an interaction term between environmental enrichment and the factor variable assigning participants to the three groups using *ILSE* (Liu et al., 2022). By performing *F-tests*, we were able to infer the significance of the interaction term.

Adjustment for covariates: Adjusted regression analyses were performed by assessing potential confounders, thereafter referred to as covariates of no interest (i.e., age, sex, educational level and socioeconomic status) as additional predictors into the FIML regression models for the entire sample and subgroups (OA, SCD, FH). We carefully selected covariates to maintain reasonable power (Field, 2005) by assessing covariates of no interest in preliminary regression analyses. Only in case of a considerable association (*p* < 0.05) of any covariate with the dependent variables (FADE-SAME scores) were regression models re-run including the respective covariates. In addition, we included scanner site (where the participant’s fMRI data was acquired) as an additional covariate to account for scanner-related variance. Multicollinearity was assessed for each FIML regression model with the variance inflation factor from the *car* package (version 3.1–2; Fox and Weisberg, 2019).

Visualization of results: Selected results of the regression analyses were visualized using a scatter plot including the respective sample and adjusting the parameters of the respective regression lines according to the output of the FIML regression with *lavaan* (Rosseel, 2012). For this purpose, we used *ggplot2* (version 3.5.1; Wickham, 2016).

## 3 Results

### 3.1 Descriptive statistics

A detailed overview of the descriptive summary statistics is provided in Table 1.

**Table 1:** Descriptive summary statistics of the DELCODE subsample.

	M	SD	Min	Max	*n*
Age	69.45	5.77	60	87	372
Sex: female/male					197/175
Subgroup: OA/SCD/FH					127/199/46
Years of education	14.91	2.90	8	20	372
ISEI score^1^	61.13	17.00	15.98	88.96	361
EE score^2^	34.19	7.93	14	55	342
Novelty FADE scores^3^ (OA/SCD/FH)	−1.43 (−1.45/−1.38/−1.56)	0.57 (0.57/0.56/0.58)	−3.60 (−2.72/−3.60/−3.19)	0.53 (0.08/0.53/−0.07)	372
Novelty SAME scores^4^ (OA/SCD/FH)	−0.34 (−0.23/−0.41/−0.31)	0.51 (0.50/0.49/0.53)	−2.85 (−1.42/−2.85/−1.42)	1.20 (1.11/0.76/1.20)	372
Memory FADE scores (OA/SCD/FH)	−0.63 (−0.64/−0.61/−0.65)	0.47 (0.48/0.48/0.37)	−2.17 (−2.17/−1.91/−1.43)	0.59 (0.50/0.59/0.26)	372
Memory SAME scores (OA/SCD/FH)	−0.98 (−0.94/−1.01/−0.95)	0.45 (0.45/0.46/0.42)	−3.39 (−2.53/−3.39/−2.13)	0.63 (0.27/0.63/0.02)	372

Descriptive summary statistics of the sample derived from the DELCODE study of *n* = 372 participants. ^1^SEI (International Socioeconomic Index of Occupational Information) scores range from 16 to 90, with higher scores corresponding to a higher socioeconomic status. A total of 11 participants were lacking data to compile respective (ISEI) scores. ^2^For 30 participants, data on various LEQ items were missing, so that the corresponding values for environmental enrichment (EE) could not be calculated. ^3^Higher FADE scores indicate higher deviation of an older adult’s brain activity patterns from those of young adults. ^4^Higher SAME scores indicate higher similarities of an older adult’s brain activation and deactivation patterns with those of young adults. Using full information maximum likelihood (FIML), we performed data analyses with the full sample of *n* = 372 (see the data processing section for a detailed description of the treatment of missing values). M, mean; SD, standard deviation; OA, cognitively unimpaired older adults; SCD, subjective cognitive decline; FH, family history of AD.

### 3.2 Confirmatory analyses

All regression assumptions regarding normality, linearity, and homoscedasticity were met.

#### 3.2.1 Results for the entire sample

**Figure 3:**
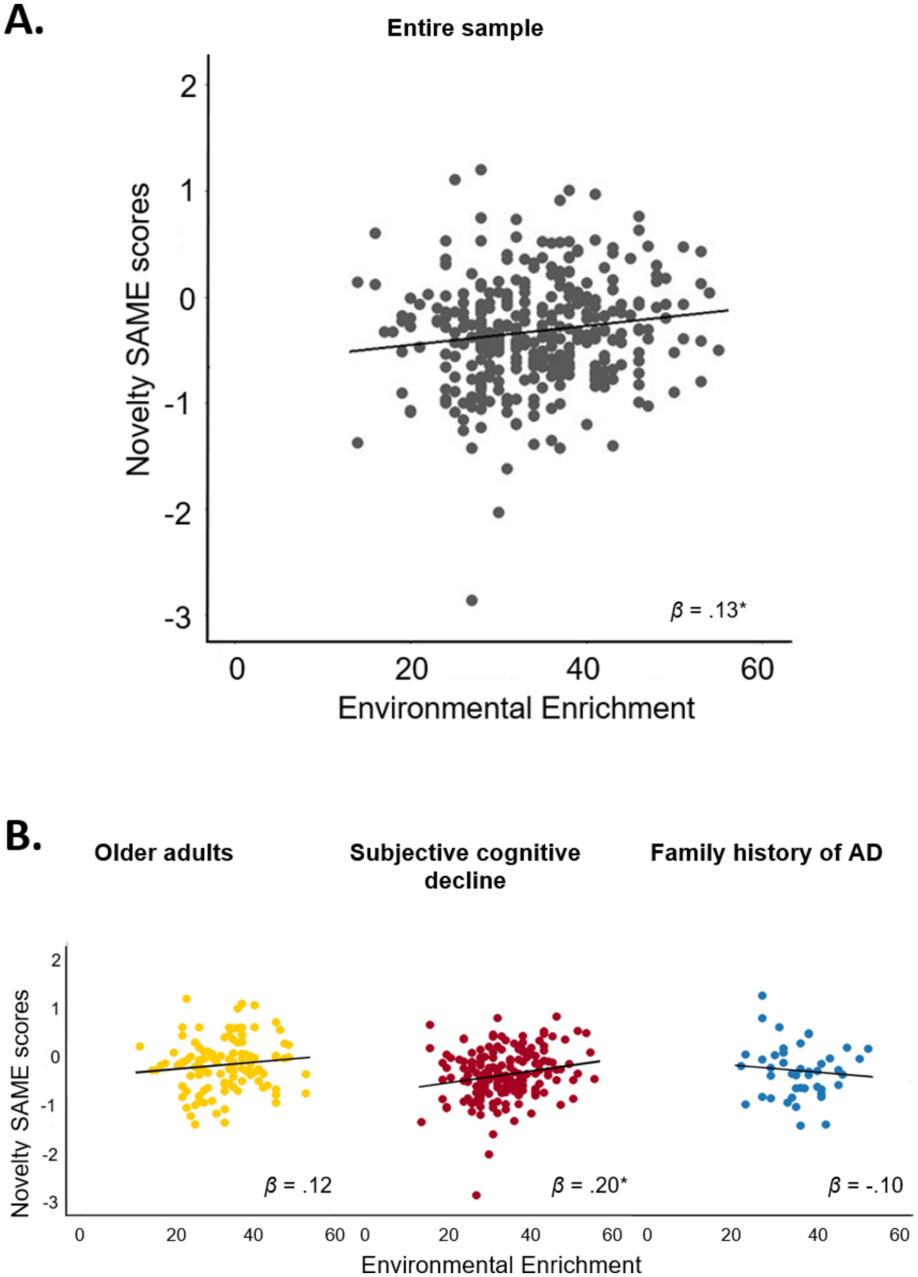
Association between environmental enrichment and SAME scores from the novelty contrast. Scatter plots display the unadjusted association between environmental enrichment and SAME scores from the novelty contrast **(A)** in the entire sample and **(B)** in the three subgroups. Environmental enrichment was significantly associated with SAME scores from the novelty contrast in the entire sample (*p* = 0.011) and in participants with subjective cognitive decline (*p* = 0.006). Since the FIML method merely estimates regression parameters, but not the missing values themselves, the 342 complete cases constitute the scattered data points; the parameters of the regression line itself (i.e., intercept and slope) are derived from the linear regression with FIML. *p < 0.05. *β*, standardized coefficient; SAME, similarity of activations during memory encoding.

Results of the regression analysis in the total sample are displayed in Table 2 and Figure 3. Environmental enrichment was significantly associated with greater SAME scores from the novelty contrast (*β* = 0.13, *p* = 0.011). Thus, participants with greater environmental enrichment showed a higher similarity of functional brain activity patterns during novelty processing to those observed in young adults. Inconsistent with our hypotheses, environmental enrichment was not associated with the remaining FADE and SAME scores (all *β* < 0.06, all *p* ≥ 0.05).

**Table 2:** Results for the simple regression analyses between memory-related functional brain activity and environmental enrichment.

Dependent variable	*n*	*B*	*SE (B)*	*β*	*p*
Analysis in the entire sample					
Novelty FADE scores^1^	372	−0.01	0.00	−0.10	0.054
Novelty SAME scores^2^	372	0.01	0.00	0.13	0.011*
Memory FADE scores	372	−0.00	0.00	−0.04	0.412
Memory SAME scores	372	0.00	0.00	0.06	0.286
Analysis within subgroups					
Novelty SAME scores; OA^3^	127	0.01	0.01	0.12	0.188
Novelty SAME scores; SCD^4^	199	0.01	0.00	0.20	0.006**
Novelty SAME scores; FH^5^	46	−0.01	0.01	−0.10	0.513

^1^Higher FADE scores indicate higher deviation of an older adult’s brain activity patterns from those of young adults. ^2^Higher SAME scores indicate higher similarities of an older adult’s brain activation and deactivation patterns with those of young adults. ^3,4,5^Coefficients for environmental enrichment within subgroups. **p < 0.01, *p < 0.05. B, unstandardized coefficient; SE (B), standard error; β, standardized coefficient referencing the respective sample (entire sample or specific subgroup); FADE, functional activity deviation during encoding; SAME, similarity of activations during memory encoding; OA, cognitively unimpaired older adults; SCD, subjective cognitive decline; FH, family history of AD.

#### 3.2.2 Results within subgroups

Results of the regression analysis within subgroups are shown in Table 2 and Figure 3. The post-hoc analyses within the three subgroups (OA, SCD, FH) showed that environmental enrichment during early life and midlife was significantly associated with greater SAME scores from the novelty contrast in participants with SCD (*β* = 0.20, *p* = 0.006). Environmental enrichment was not associated with SAME scores from the novelty contrast in OA and participants with FH (all *β* < 0.13, all *p* > 0.19).

To further explore whether group classification would affect the association between environmental enrichment and the SAME scores from the novelty contrast, we compared a model with an interaction term between diagnostic groups (OA, SCD, FH) and environmental enrichment and a model without using an *F-test*. Group classification had no significant effect on the regression slopes on a group level with *F*_(4, 194)_ = 2.67, *p* = 0.070.

#### 3.2.3 Results of covariate adjustment

Results for the covariate-adjusted regression analyses are shown in Table 3. Of all the considered covariates of no interest, age and sex showed considerable associations with the FADE and SAME scores (all *p* < 0.05). When adding these potential confounders, environmental enrichment during early life and midlife was significantly associated with greater SAME scores from the novelty contrast in the entire sample (*β* = 0.11, *p* = 0.036). There were no significant associations between environmental enrichment in early life and midlife for the remaining FADE and SAME scores after covariate adjustment (all *β* < 0.04, all *p* > 0.23).

**Table 3:** Results for the covariate-adjusted regression analyses between memory-related functional brain activity and environmental enrichment.

Dependent variable	*n*	*B*	*SE (B)*	*β*	*p*
Analysis in the entire sample					
Novelty FADE scores^1^	372	−0.01	0.00	−0.06	0.230
Novelty SAME scores^2^	372	0.01	0.00	0.11	0.036*
Memory FADE scores	372	−0.00	0.00	−0.00	0.980
Memory SAME scores	372	0.00	0.00	0.03	0.541
Analysis within subgroups					
Novelty SAME scores; OA^3^	127	0.01	0.01	0.15	0.089
Novelty SAME scores; SCD^4^	199	0.01	0.00	0.16	0.029*
Novelty SAME scores; FH^5^	46	−0.01	0.01	−0.11	0.443

All models are adjusted for age and sex. ^1^Higher FADE scores indicate higher deviation of an older adult’s brain activation patterns from those of young adults. ^2^Higher SAME scores indicate higher similarities of an older adult’s brain activation and deactivation patterns with those of young adults. ^3,4,5^Coefficients for environmental enrichment within subgroups. *p < 0.05. B, unstandardized coefficient; SE (B), standard error; β, standardized coefficient referencing the respective sample (entire sample or specific subgroup); FADE, functional activity deviation during encoding; SAME, similarity of activations during memory encoding; OA, cognitively unimpaired older adults; SCD, subjective cognitive decline; FH, family history of AD.

Post-hoc analyses within subgroups (OA, SCD, FH) showed that environmental enrichment was significantly associated with the SAME scores from the novelty contrast in participants with SCD after adjustment for age and sex as potential confounders (*β* = 0.16, *p* = 0.029). There were no significant associations between environmental enrichment and SAME scores from the novelty contrast in neither OA nor participants with FH (all *β* < 0.16, all *p* > 0.08).

When adding fMRI recording site as a categorical factor to the model, the main associations between environmental enrichment and SAME scores from the novelty contrast were maintained for the entire sample (*β* = 0.11, *p* = 0.043) and within the SCD subgroup (*β* = 0.15, *p* = 0.029).

## 4 Discussion

The present cross-sectional study took a novel approach by investigating the association between self-reported environmental enrichment during early life and midlife (as measured by the frequency of participation in a variety of leisure activities) and the preservation of memory-related functional brain activity patterns (as measured by FADE and SAME scores—essentially biomarkers for the successful aging of the memory network). By considering a broad range of leisure activities, our study expands on previous research that has predominantly focused on physical activity as a predictor of successful brain aging. The study was conducted in a sample of cognitively unimpaired older participants of the DELCODE study (Jessen et al., 2018). We found that older adults who reported greater environmental enrichment during life showed greater similarity of their functional brain activity patterns during novelty processing to the functional brain activity patterns observed in younger adults. This association was found most strongly in participants with SCD. The findings suggest that more frequent participation in a variety of leisure activities may relate to better preservation of memory-related functional brain activity in older adults. Longitudinal studies are needed to determine whether environmental enrichment across the lifespan could facilitate successful aging of memory network function in later life.

### 4.1 Environmental enrichment and successful brain aging

As a novel finding, this study demonstrates that higher levels of environmental enrichment (i.e., participation in social, musical, artistic and physical activities, as well as reading and speaking a second language) during early life and midlife were associated with higher SAME novelty scores in the older participants of the DELCODE cohort. This specific single-value score reflects the similarity of novelty-related functional brain activity patterns to the prototypical pattern found in the temporo-parieto-occipital memory network in young adults (Soch et al., 2021, 2024). Consequently, a higher SAME score in older adults may be interpreted as a biomarker of successful aging of memory network function (Düzel et al., 2010). Consistent with our findings, a recent study in an independent cohort of healthy older adults showed that openness to experience, a personality trait associated with environmental enrichment (Trapp et al., 2019), was associated with more prototypical memory-related functional brain activity (i.e., higher SAME scores and lower FADE scores; Stolz et al., 2023).

It has been proposed that environmental enrichment across the lifespan may serve as a strategy to maintain or promote brain health in older age and potentially alter individual susceptibility to developing dementia (Kempermann, 2022). Although the present cross-sectional findings do not permit the inference of causality, it appears that greater environmental enrichment during early life and midlife is linked to more successful aging of memory network function in older adults. This finding aligns with and extend previous results in human studies that have demonstrated associations between environmental enrichment and brain health indicators, including brain volume (Anatürk et al., 2018; Duffner et al., 2023), hippocampal volume (Duffner et al., 2023), and fornix microstructure integrity (Klimecki et al., 2023). Moreover, our recent studies have shown that lifelong musical activity, as a multimodal enrichment strategy, is associated with more efficient use of brain capacity (Böttcher et al., 2022) and higher functional connectivity (Liebscher et al., 2024) in distributed brain regions. Taken together, these findings suggest that environmental enrichment may have multiple benefits for brain health in later life, manifesting at structural and functional levels of the brain. The question remains as to whether this phenomenon is indicative of mechanisms of cognitive reserve and resilience (Stern et al., 2020).

The association between environmental enrichment and a higher SAME novelty score was particularly evident in older participants with SCD. Studies have suggested that SCD is a known risk factor for the development of cognitive impairment and dementia (Jessen et al., 2010). Interestingly, in a previous study of the DELCODE cohort, the SAME novelty score was found to be particularly sensitive to AD risk factors (Soch et al., 2024). Older adults with SCD had lower SAME novelty scores at baseline compared with control participants, whereas no differences were found between the groups for FADE novelty scores. In the same study, the SAME novelty score indicated the presence or absence of AD pathology, such as the amyloid beta (Aβ) status as measured by cerebrospinal fluid (CSF). It remains unclear as to why the association between environmental enrichment and novelty SAME scores was mainly observed in the SCD group. The presence of early-stage brain injury in older adults with SCD (e.g., Wirth et al., 2018; Schwarz et al., 2022) may increase inter-individual variability and reliance on reserve mechanisms. The SCD sample was also larger than the other samples (OA, FH), which allowed for greater statistical power. Future research is needed to assess whether environmental enrichment can help preserve memory network function in older adults at risk of dementia.

In general, the present findings extend previous research showing that higher levels of physical activity are associated with enhanced/preserved functional brain activity related to memory in older adults (Domingos et al., 2021) in several ways: first, we included a large variety of musical, artistic, social, and cognitive leisure activities as opposed to just physical activity (Smith et al., 2016; Thielen et al., 2016). Second, we linked engagement in several leisure activities to functional activity patterns in the human memory network, which includes not only the hippocampus, but the dorsolateral prefrontal cortex and temporo-parieto-occipital cortices (Soch et al., 2021). Given the past and current findings of a positive association between environmental enrichment and brain structure/function, it appears that engaging in a variety of leisure activities throughout life may help preserve the memory network in later life. In the context of embodied prevention (Kempermann, 2022), it remains to be shown which intervention is most effective to promote healthy aging of the mind and brain: physical, cognitive, social or more integrated/holistic activities such as music-making and dancing (e.g., Böttcher et al., 2022; Podolski et al., 2023). A recent meta-analysis further demonstrated that combined interventions (i.e., physical *and* cognitive activities) had superior effects over single physical exercise on memory and attention (Meng et al., 2022), but more research is needed to substantiate these findings.

### 4.2 Perspectives: FADE and SAME scores as proxies for neurocognitive aging

In a broader perspective, the specific associations between the four memory-related brain activity scores (FADE vs. SAME score * novelty vs. subsequent memory contrast) and numerous factors affecting cognitive aging and different cognitive domains should be considered. Earlier findings in an independent cohort of healthy middle-aged and older adults have suggested that novelty-related scores are primarily associated with performance in hippocampus-dependent memory tasks, whereas memory-related scores are additionally associated with more global measures of cognition (Richter et al., 2023). Beyond that, the novelty-related scores have been recently linked to lower CSF-measured AD pathology in individuals with SCD as well as in healthy relatives of AD patients (Soch et al., 2024). Thus, we have proposed that the novelty-related scores may provide information about neurocognitive risk and reserve in older adults at increased risk for dementia. In contrast, the memory-related scores may be more informative with respect to cognitive functioning in healthy older adults (for a comprehensive discussion, see Soch et al., 2024).

Apart from the distinction between novelty-related and memory-related scores, a potentially differential utility of the various score types (FADE vs. SAME) needs to be addressed. Both, FADE and SAME scores have been proposed to reflect measures of successful aging of the memory network (Düzel et al., 2010; Soch et al., 2021, 2024; Richter et al., 2023; Stolz et al., 2023). As an important difference between these scores, the SAME scores include functional deactivation patterns that are related to novelty and memory processing, respectively, (Soch et al., 2021). Interestingly, young adults show pronounced Default Mode Network (DMN) deactivations during novelty processing and successful encoding that are attenuated with aging (Kim, 2011; Maillet and Rajah, 2014; Schott et al., 2023). The deactivations in the DMN during novelty processing are further reduced in older adults with SCD, while functional activations in the temporo-parieto-occipital memory network are relatively preserved (Billette et al., 2022; Soch et al., 2024). The differences between healthy controls and risk groups in the SAME novelty score and the association between environmental enrichment and the SAME novelty score may be related to the importance of functional deactivations in neurocognitive aging (Maillet and Schacter, 2016).

### 4.3 Limitations and directions for future research

The present study has several strengths. We gathered cross-sectional data from the observational multicenter DELCODE cohort to assess a large and well-characterized sample of cognitively unimpaired adults (aged 60 years and older) with self-reported engagement in a variety of leisure activities during early life and midlife. All measures, including FADE-SAME scores, were collected using harmonized acquisition protocols and high-quality (neuroimaging) data assessments across assessment sites. We further generated new evidence on the potential benefits of leisure activities on memory network function in older adults.

Some limitations regarding the results of this research and derived implications need to be addressed. Due to the cross-sectional nature of the baseline data, causality cannot be inferred. Furthermore, our findings cannot be generalized to patients with MCI, AD, or other types of dementia since we only included cognitively unimpaired older adults in our sample. Longitudinal intervention studies with more heterogeneous samples are thus required. In addition, the reference sample (i.e., 106 young adults) that was recruited for the computation of the FADE/SAME scores (Soch et al., 2021, 2024) differed from the older adults of the DELCODE subsample in terms of their educational background (i.e., ~47% of the older adults, but ~94% of the young adults had obtained the Abitur, the German equivalent of a high school diploma). This difference is likely not substantial, however, since the percentage of people attaining the “Abitur” in Germany has increased considerably over the past decades, the overall educational attainment of the older participants was likely above the average of their respective generation. This was previously reported in an independent, but demographically comparable cohort (for discussion, see Soch et al., 2021; Richter et al., 2023).

Overall, future studies will be necessary to assess whether our findings are representative of and generalizable to the broader population. Although the LEQ is a well-established and validated questionnaire (Valenzuela and Sachdev, 2007), retrospective self-reports of the participation in leisure activities may be biased, e.g., by social desirability effects or distorted self-perception. Such biases could perhaps be avoided with more objective measures or close monitoring in longitudinal studies. The present sample primarily consisted of individuals from Western, educated, industrialized, rich, and democratic countries (WEIRD; Henrich et al., 2010). An important reason for this may have been that sufficient knowledge of German was one of the inclusion criteria. Additionally, the recruiting strategy based on memory clinics and newspaper advertisements may have contributed to a potential selection bias, as individuals from minority groups have been reported to be underrepresented in memory clinics (Hinton et al., 2024). Furthermore, several participating centers recruited their participants solely or partly from East Germany, where only few individuals from minority populations lived prior to 1989. Therefore, the relative ethnic and social homogeneity of our study sample must be considered a limitation, and replication in ethnically and culturally diverse populations is desirable (Dotson and Duarte, 2020). Comparative investigations with longitudinal studies in other geographical regions like Canada (Tremblay-Mercier et al., 2021) or China (Shao et al., 2024) may help to clarify reproducibility and generalizability of findings from the DELCODE study. Finally, to establish causality, longitudinal research will be needed to determine the relationship between environmental enrichment and the maintenance of memory-related functional brain activity in older age.

## 5 Conclusion

The results of this cross-sectional study in cognitively unimpaired older adults show that environmental enrichment, as measured by the self-reported participation in a variety of leisure activities during early life and midlife, is associated with greater similarity of functional brain activity patterns in the memory network to those observed in young adults. To establish causality, future research is needed to determine whether environmental enrichment across the lifespan could help preserve memory network function in later life.

## Data Availability

The data analyzed in this study is subject to the following licenses/restrictions: the anonymized data used for this study will be made available by request from any qualified investigator through the DZNE-DELCODE Steering Board for purposes of replicating procedures and results. Requests to access the minimal dataset should be directed to the German Center for Neurodegenerative Diseases (DZNE), Bonn. For contact information please refer to: https://www.dzne.de/en/research/studies/clinical-studies/delcode/ (Study Coordination and Project Management).
