# Supplementary material for: Environmental enrichment is associated with favorable memory-related functional brain activity patterns in older adults
Source: Front Aging Neurosci. 2024 Dec 24;16:1451850. doi: 10.3389/fnagi.2024.1451850 (PMC11704887; doi:10.3389/fnagi.2024.1451850)
Supplement: Supplementary file 1 [file Table_1.DOCX]

# **Supplementary Material**

**Supplement A1: Acquisition of fMRI Data**

MRI data were acquired at eight different sites across Germany using Siemens 3T MR tomographs. All sites followed the DELCODE MRI protocol (Soch et al., 2021). Structural MRI included a T1-weighted MPRAGE image (TR = 2.5 s, TE = 4.37 ms, flip-α = 7°; 192 slices, 256 × 256 in-plane resolution, voxel size = 1 × 1 × 1 mm) for co-registration and improved spatial normalization. Phase and magnitude fieldmap images were acquired to improve correction for artifacts resulting from magnetic field inhomogeneities***.*** Functional MRI consisted of 206 T2*-weighted echo-planar images (EPIs; TR = 2.58 s, TE = 30 ms, flip-α = 80°; 47 slices, 64 × 64 in-plane resolution, voxel size = 3.5 × 3.5 × 3.5 mm) and a resting-state session (180 scans, same parameters, not used here) (Soch et al., 2024).

**Supplement A2: Processing of fMRI Data**

Data processing and analysis were performed using Statistical Parametric Mapping, version 12 (SPM12; Wellcome Centre for Human Neuroimaging, University College London, London, UK; https://www.fil.ion.ucl.ac.uk/spm/software/spm12/) and in-house MATLAB scripts (https://github.com/JoramSoch/FADE_SAME). Preprocessing of fMRI data included correction for acquisition time (slice timing), head motion (realignment), and magnetic field inhomogeneities using the fieldmaps (unwarping), coregistration of the T1-weighted MPRAGE image to the mean EPI computed during realignment, segmentation of the coregistered MPRAGE image, subsequent normalization of unwarped EPIs into the MNI standard space (voxel size = 3 × 3 × 3 mm), and spatial smoothing of the normalized EPIs (FWHM = 6 mm). Statistical analysis of the fMRI data was based on voxel-wise general linear models (GLMs) that included two onset regressors, representing novel images (novelty regressor) and pre-familiarized images (master regressor), six head motion regressors obtained from realignment and a constant representing the implicit baseline. The novelty regressor was parametrically modulated with the arcsine-transformed subsequent memory response, yielding a regressor reflecting encoding success (Soch et al., 2024).

**Supplement A3: Calculation of FADE-SAME scores**

Young adults’ activated networks of brain regions in either the novelty processing or the subsequent memory contrast were used as reference maps. For this purpose,106 young adults between 18 and 35 years (mean age 24.12 ± 4.00 years, 44% males; Soch et al., 2021) had been recruited from universities. Reference maps were generated by performing group-level statistical tests identifying sets of voxels with significant positive effects (novelty: novel > master; memory: higher effect for remembered items) or negative effects (novelty: novel < master; memory: higher activations for forgotten items). Calculation of FADE-SAME scores was then done as follows: The FADE score equals the mean *t*-value on a contrast (e.g., the novelty contrast) for an older adult, averaged over all voxels in which the young adults showed a positive effect on this contrast (e.g., for the novelty contrast: higher activations for items that are novel), subtracted from the average t-value in all the other voxels. The SAME score is equal to the mean reduced activation of an older adult (e.g., during novelty processing) averaged over all voxels in which the young adults activated (e.g., during novelty processing), plus the mean reduced deactivation averaged over all voxels in which the young adults deactivated (e.g., for the novelty contrast: higher deactivations for old items), weighted by each voxel’s standard deviation of the young adults (see Figure 1 and Appendix A in Soch et al., 2021).
